# The complete chloroplast genome sequence of a medicinal citrus landrace, *Citrus erythrosa* Hort. ex Tanaka in Jeju Island, Korea

**DOI:** 10.1080/23802359.2022.2057243

**Published:** 2022-04-01

**Authors:** Seung Chul Shin, Ji Hoon Song, Yo-Han Yoo, Joo-Sang Lee, Seong-Il Kang, Hee Jeong Kim, Hyoungseok Lee, Ho Bang Kim

**Affiliations:** a Korea Polar Research Institute, Incheon, Republic of Korea; b Jeju Institute of Korean Medicine, Jeju, Republic of Korea; c Polar Science Department, University of Science & Technology, Incheon, Republic of Korea; d Life Sciences Research Institute, Biomedic Co., Ltd., Bucheon, Republic of Korea

**Keywords:** *Citrus erythrosa*, landrace, medicinal fruit, chloroplast genome, phylogenetic analysis

## Abstract

*Citrus erythrosa* (Dongjeongkyool in Korean) is a medicinal citrus landrace that grows in Korea. In this study, we characterized the complete chloroplast (Cp) genome (160,120 bp) of* C. erythrosa*. The Cp genome was consisted of 4 distinct regions: a large single copy (87,731 bp), a small single copy (18,393 bp), and a pair of inverted repeat regions (26,998 bp). The Cp genome encodes a total of 133 genes including 88 protein-coding genes, 37 tRNA genes and 8 rRNA genes. The phylogenetic analysis reveals that *C. erythrosa* is a sister group to the clade of species including *C. reticulata* within the genus *Citrus.*

The genus *Citrus* which includes mandarins, oranges, grapefruits, lemons, and limes has high economic and nutritional values. This genus belongs to the subfamily Aurantioideae of the Rutaceae family, which consists of 2 tribes (Clauseneae and Citreae) with 33 genera and over 1700 species. The genus *Citrus* and its related genera such as *Eremocitrus*, *Fortunella*, *Microcitrus*, and *Poncirus* belong to the ‘true citrus fruit trees’ of the Citreae tribe (Swingle and Reece 1967). According to biochemical studies, numerical taxonomy, and recent molecular phylogenetic studies, all cultivated *Citrus* species are believed to result from interspecific hybridization between four fundamental or primary species [citron (*Citrus medica* L.), mandarin (*Citrus reticulata* Blanco), pummelo (*Citrus maxima* Burm. Merrill), and papeda (*Citrus micrantha* Wester)] (Nicolosi 2007; Curk et al. 2016).

Zeven (1998) proposed that landrace is a variety with a high capacity to tolerate biotic and abiotic stresses, emphasizing the importance of landraces as genetic resources. 13 citrus landraces grow in Jeju, South Korea, including *Citrus erythrosa* Hort. ex Tanaka (Tanaka 1927; IPNI 2021), which is called ‘Dongjeongkyool’ in Korean and is a mandarin landrace (Park et al. 2020). Fruit peel and young fruit of many citrus species including *C. erythrosa* have long been used as traditional Asian medicine ingredients due to their gastric protection, antiulcer, cholesterol-lowering, and anti-cancer effects (Zhang et al. 2007). Here we assembled and annotated the complete chloroplast (Cp) genome sequence of *C. erythrosa* growing in Jeju island, to provide a basic genetic resource for *Citrus* hybrids with other species in the genus *Citrus*.

Fresh leaves of *C. erythrosa* (the voucher number IT233658) were obtained from an experimental station of Agricultural Research and Extension Services, Jeju Special Self-Governing Province, Republic of Korea (33°15′39″N/126°29′21″E). Total genomic DNA was purified using Biomedic^®^ Plant gDNA Extraction Kit (Biomedic Co., Ltd., Bucheon, Korea) according to the manufacturer’s protocol. A genomic library for Illumina sequencing was constructed using the TrueSeq Nano DNA library kit (Illumina, Inc., San Diego, CA, USA) with an average fragment size range of 500 bp. The constructed library was sequenced using the Illumina NovaSeq6000 (151PE). All sequencing procedures were performed by Phyzen Co. Ltd. (Seongnam, Korea). A total of 34,992,300 Illumina reads comprising 5.3 Gbp were generated. To trim Illumina reads by quality and remove the adapters, Trimmomatic version 0.38 (Bolger et al. 2014) was applied with the following parameters: ‘ILLUMINACLIP:adaptor.file:2:30:10 LEADING:3 TRAILING:3 SLIDINGWINDOW:4:15 MINLEN:36’. Trimmed reads were mapped to the publicly available Cp genome of *Citrus* species; *C. aurantiifolia* (NC_024929.1), *C. depressa* (NC_031894.1), *C. limon* (NC_034690.1), *C. maxima* (NC_034290.1), *C. platymamma* (NC_030194.1), *C. reticulata* (NC_034671.1), and *C. sinensis* (NC_008334.1) using Bowtie2 v.2.3.4 (Langmead et al. 2009), in order to select putative Cp reads, and the resulting aligned reads were assembled into contigs using ABySS (v2.1.5) (Simpson et al. 2009). Assembled Cp contigs were aligned to the reference Cp genome with the highest identity and were concatenated into larger contigs using CLC Genomics Workbench v7 by joining overlapping terminal sequences. The genome was annotated by GeSeq (Tillich et al. 2017), followed by manual confirmation using BLAST searches. A specimen was deposited at Agricultural Research and Extension Services, Jeju Special Self-Governing Province, Republic of Korea (URL https://agri.jeju.go.kr/agri/index.htm, contact person: Mr. Sang Hoon Kang, e-mail: kshjeju@korea.kr) under the voucher number IT233658.

The complete Cp genome of *C. erythrosa* (GenBank Accession No. MW722946) was 160,120 bp in length and composed of four distinct regions such as a large single copy (LSC) (87,731 bp), a small single copy (SSC) (18,393 bp), and a pair of inverted repeats (IRa and IRb) (each 26,998 bp). A total of 133 genes including 88 protein-coding genes, 37 tRNA genes, and 8 rRNA genes was identified in the Cp genome. The aligned complete Cp genome sequences of *C. erythrosa* and those of 11 Aurantioideae species were used for phylogenetic analysis by MEGA-X (Kumar et al. 2018). The phylogenetic tree was constructed using the maximum likelihood method with 1000 bootstrap replications. The tree shows that *C. erythrosa* is a sister group to the clade of 6 species including *C. maxima* (pummelo) and *C. reticulata* (mandarin) (Figure 1). The tree topology was similar to the tree inferred from cytogenetic analysis of Korean landrace citrus (Yi et al. 2018). The complete Cp genome sequence of *C. erythrosa* will be valuable information for DNA super-barcoding and phylogenetic origin analysis of the genus *Citrus* (Li et al. 2015; Curk et al. 2016).

**Figure 1.**
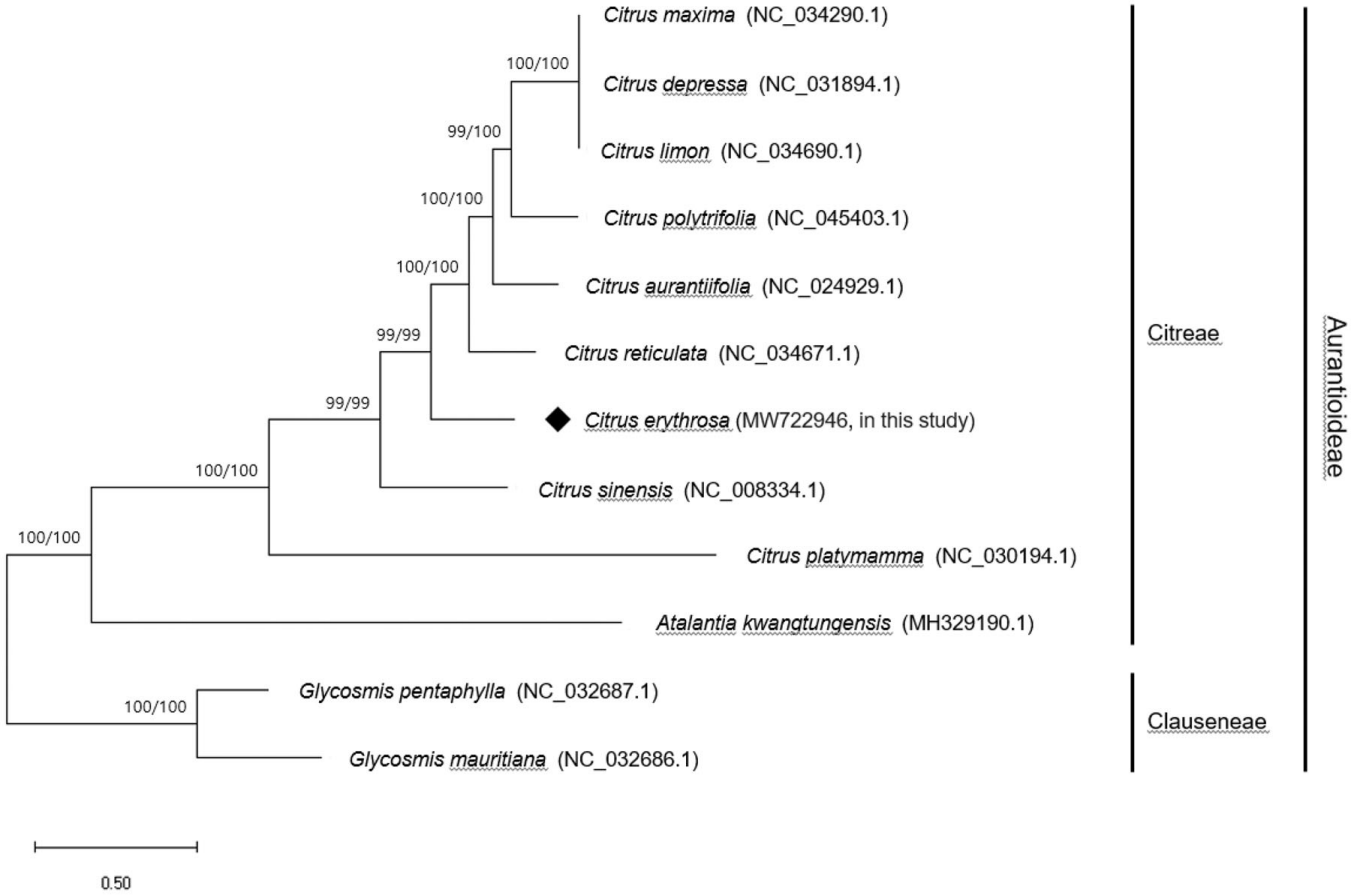
Maximum-likelihood (ML) tree based on the complete chloroplast genome sequences of *C. erythrosa* and 11 Aurantioideae species. Bootstrap analyses were conducted in ML and NJ with 1000 replicates and values of ML/NJ are shown on branches.

## Authors’ contributions

Seung Chul Shin: Investigation, Ji Hoon Song: Investigation, Yo-Han Yoo: Investigation, Joo-Sang Lee: Funding acquisition, Conception & design, Seong-Il Kang: Writing-review & editing, Data interpretation, Hee Jeong Kim: Funding acquisition, Hyoungseok Lee: Writing-original draft, Data interpretation, Ho Bang Kim: Writing-review & editing, Funding acquisition, Conception & design, Supervision.

## Data Availability

The genome sequence data that support the findings of this study are openly available in GenBank of NCBI at (https://www.ncbi.nlm.nih.gov/) under the accession no. MW722946. The associated BioProject, SRA, and Bio-Sample numbers are PRJNA783531, SRP347861, and SAMN23437889, respectively.
